
JOURNAL INFORMATION
==============================
NLM Title Abbreviation: Buffalo Med J
NLM Journal ID: 0027534

Buffalo Medical Journal

ISSN: 1040-3817
Publisher: Biomedical Journal Digitization Project

ARTICLE INFORMATION
==============================
PMCID: PMC8726788
PMID: 36885224
Article version: 1
Subjects: Item

Item

Print publication date: 1909 Mar
Volume: 64
Issue: 8
First page: 468
Last page: 468
License: This work is free of known copyright restrictions. For more information, please see PMC Back Issue Digitization.
License URL: https://creativecommons.org/publicdomain/mark/1.0/

==============================

### LITERARY NOTES

SAUNDERS'S Books is the title of a new descriptive catalogue of medical and surgical works issued by the W. B. Saunders Company, medical publishers, Philadelphia and London. It is handsomely printed in tinted paper, fully illustrated, and is a beautiful specimen of the printing and engraving arts. It contains a description of almost every book a physician really reads and may be obtained on application to the publishers, 925 Walnut Street, Philadelphia.

---

THE *American Journal of Surgery,* New York, announces that Dr. James P. Warbasse, late editor N. Y. State Medical Journal, has joined its editorial staff. The March number will contain articles by Lilienthal, Young, Simpson, Hopkins, Tuttle, Brickner, Warbasse, Bandler, Meyer, Hartwell, deLatour, Sellings, and others.

---

### ITEM.

BATTLE & Co., 2001 Locust Street, St. Louis, Mo., have issued No. 8 of their Dislocation Chart series. Physicians desiring any back numbers can get them upon request to the publishers.

### WANTED: MEDICAL EDITING and PROOFREADING

MEDICAL EDITING AND PROOFREADING desired by a woman well qualified. Articles prepared from notes. All details looked after for busy physicians. Years of experience, with technical knowledge of medicine, English and several other languages. Have done work for eminent men and on the most successful text-books. Prices reasonable.

Address

BUFFALO MEDICAL JOURNAL

238 DELAWARE AVENUE, BUFFALO, N. Y.
